# Perspectives on optimizing local delivery of drugs to peripheral nerves using mathematical models

**DOI:** 10.1002/wsbm.1593

**Published:** 2023-01-09

**Authors:** Simao Laranjeira, Victoria H. Roberton, James B. Phillips, Rebecca J. Shipley

**Affiliations:** ^1^ UCL Mechanical Engineering UCL Centre for Nerve Engineering London London UK; ^2^ UCL School of Pharmacy UCL Centre for Nerve Engineering London London UK

**Keywords:** local drug delivery, mathematical modelling, multidisciplinary approach, peripheral nerve repair

## Abstract

Drug therapies for treating peripheral nerve injury repair have shown significant promise in preclinical studies. Despite this, drug treatments are not used routinely clinically to treat patients with peripheral nerve injuries. Drugs delivered systemically are often associated with adverse effects to other tissues and organs; it remains challenging to predict the effective concentration needed at an injured nerve and the appropriate delivery strategy. Local drug delivery approaches are being developed to mitigate this, for example via injections or biomaterial‐mediated release. We propose the integration of mathematical modeling into the development of local drug delivery protocols for peripheral nerve injury repair. Mathematical models have the potential to inform understanding of the different transport mechanisms at play, as well as quantitative predictions around the efficacy of individual local delivery protocols. We discuss existing approaches in the literature, including drawing from other research fields, and present a process for taking forward an integrated mathematical‐experimental approach to accelerate local drug delivery approaches for peripheral nerve injury repair.

This article is categorized under:

Neurological Diseases > Molecular and Cellular Physiology
Neurological Diseases > Computational Models
Neurological Diseases > Biomedical Engineering

## Graphical abstract

Numerous local drug delivery approaches are being developed to treat peripheral nerve injuries and to avoid adverse effects induced by systemic delivery. Exploring and optimizing the numerous delivery strategies using an experimental approach in isolation has proven to be a barrier to innovation. Here, a novel framework is proposed, where in silico and experimental models are devised in parallel.

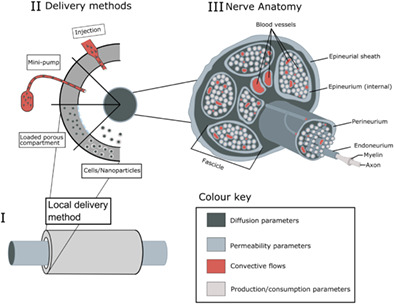

## 1 INTRODUCTION

The peripheral nervous system (PNS) contains nerves that transmit motor and sensory signals between peripheral organs and the central nervous system (CNS). Injuries to these nerves are common, affecting millions of people per year globally with the most severe injuries requiring surgical repair (Palispis & Gupta, 2017). The PNS has regenerative potential, though the slow rate of regeneration can inhibit functional recovery. Loss of innervation can cause the target organ to atrophy irreversibly and patients may experience pain and disability (Palispis & Gupta, 2017), with high economic, healthcare and workforce implications. Current therapies involve pain management, rehabilitation, and surgical intervention for more severe injuries. Drug treatments are not used routinely to accelerate regeneration and improve functional outcomes.

A number of drugs have, however, shown promise preclinically for improving regeneration after peripheral nerve injury (PNI) including small molecules, immunosuppressants, and growth factors (Bota & Fodor, 2019; Rayner, Healy, & Phillips, 2020). These drugs are typically delivered systemically; translation to the clinic has been hindered by the challenge of achieving an effective concentration at the injured nerve, without causing adverse effects to other tissues and organs. Local drug delivery could mitigate this, for example through delivery adjacent to the nerve via injection or release from biomaterials. However, it remains unclear how to relate the dose required at the molecular target within the nerve to that delivered. Currently this relationship is explored via extensive in vitro and in vivo experiments to establish the pharmacokinetics of local drug delivery and optimal dosing regimens. We propose the use of mathematical modeling to streamline this process. Mathematical models take the key geometrical, physical, and pharmacokinetic parameters as inputs and predict the temporal and spatial distribution of a drug. To be predictive, the input parameters must be characterized through carefully tailored experiments. The models can then provide insight into the dominant mechanisms at play (e.g., does diffusion dominate over decay for a particular compound), and predictions of the models can inform dosing strategies and the development of new local delivery technologies. Mathematical modeling for drug delivery has been used in other applications but has not yet been applied to peripheral nerve repair.

First, we present key features of peripheral nerve anatomy, injury and repair in the context of local drug delivery. Next, we discuss the potential offered through local drug delivery for PNI repair. We introduce the opportunity for mathematical modeling to inform local delivery strategies, providing examples from other fields where drug transport modeling is standard as well as examples, where they exist, of relevant approaches for PNI. Finally, we propose a combined approach of modeling and experimentation to accelerate the development of local drug delivery technologies for PNI repair.

## 2 PERIPHERAL NERVE ANATOMY, INJURY, AND REPAIR

### 2.1 Peripheral nerves: Anatomy and injury

**FIGURE 1:**
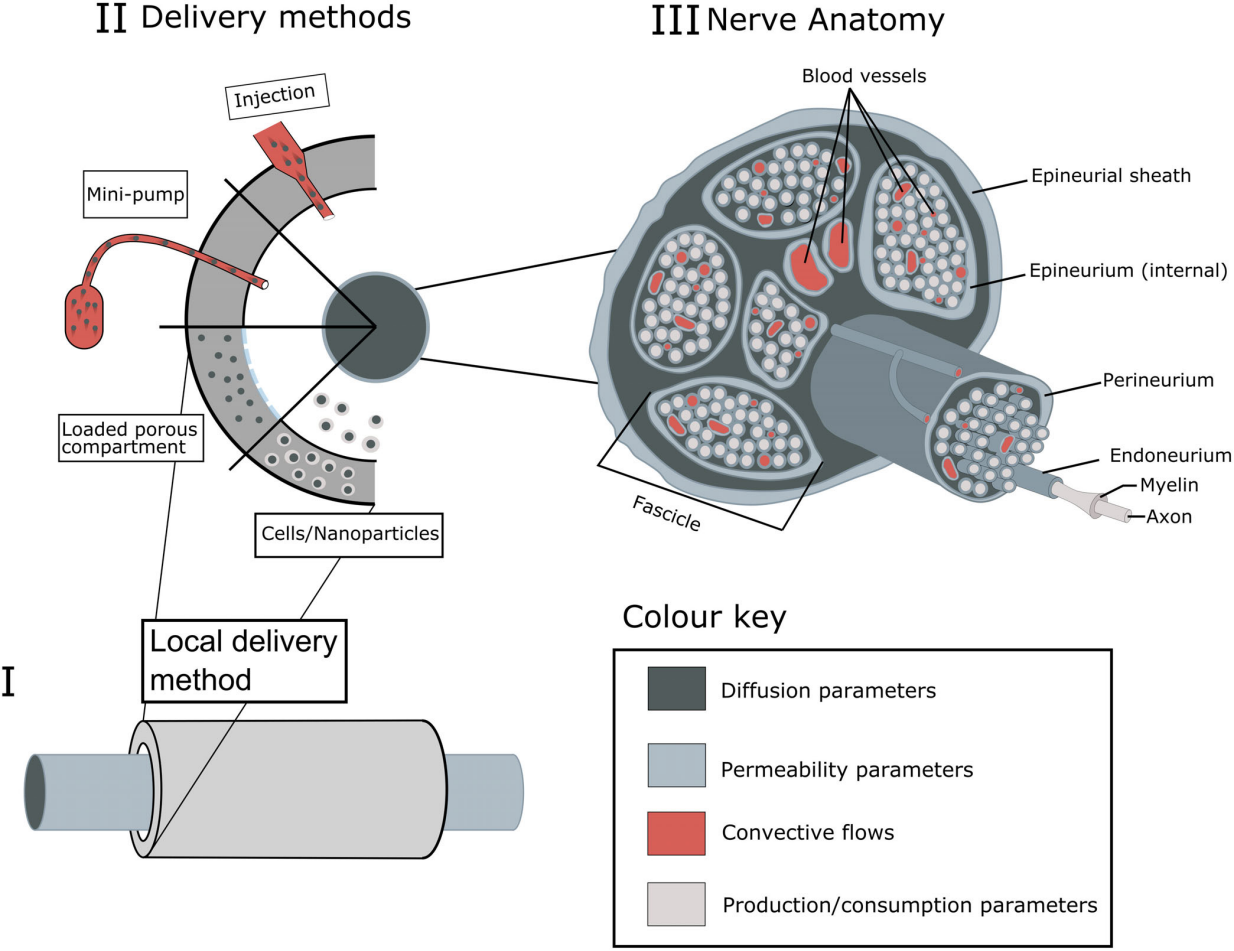
A schematic representation of the different methods of drug delivery locally in peripheral nerve injury repair (a, b) and the anatomy of peripheral nerves (c)

The architecture of a peripheral nerve is summarized in Figure 1 and constitutes a rich, hierarchical structure with cellular and matrix components. The key functional component in peripheral nerves are the bundles of axons: long thin structures that extend from the CNS to the peripheral organs and conduct either motor or sensory signals (Bota & Fodor, 2019). Individual axons are ensheathed by Schwann cells, embedded within the endoneurium and together arranged in bundles known as fascicles surrounded by the perineurium. Bundles of fascicles are encased in an outer epineurium, a collagenous tissue layer which provides structural support (Langert & Brey, 2018; Peltonen et al., 2013; Standring, 2020). Blood is supplied to the peripheral nerves via a microvascular network which penetrates (and connects across) all layers of the nerve, as described in (Lundborg, 1988).

The transport of substances into the endoneurium is usually regulated by two barriers; the perineurial nerve barrier and the endoneurial blood nerve barrier (BNB). These barriers separate the contents of the endoneurium from the surrounding tissue, limiting passage of substances through tight junctions between cells in the perineurium and lining the endoneurium wall (Azzam et al., 1991; Peltonen et al., 2013). The perineurium maintains homeostasis of the endoneurium, including intrafascicular pressure, by forming a metabolically active diffusion barrier. This prevents substances from diffusing into axon bundles, thus providing protection from toxicity and infection (Hirasawa et al., 1994; Kristensson & Olsson, 1971; Peltonen et al., 2013; Zalewski et al., 1993). This barrier is maintained through three components, the tight junctions between perineurial cells, the basement membranes surrounding perineurial cell layers, and active transcytotic transport through the perineurial cells via specific membrane receptors (Allt & Lawrenson, 2000). Following injury, recovery of the perineurium is led by proliferation of fibroblasts, which gradually acquire the morphology of perineurial cells and surround the regenerating nerve fibers, forming small funiculi (Hirasawa et al., 1994). Immediately after injury, perineurial permeability is increased, though over time the barrier function is returned. The formation of perineurial sheaths around axonal mini fascicles only consisting of one or two cell layers can still function effectively as a permeability barrier, as with a multi‐layered perineurium (Azzam et al., 1991).

The BNB regulates movement of substances from endoneurial blood vessels into the endoneurium, also via impermeable intercellular junctions (Zalewski et al., 1993). Similar to the blood brain barrier, the specialized tight junctions linking endothelial cells and pericytes of the endoneurial vasculature prevent molecules and ions in the systemic circulation from interfering with signal transduction along motor and sensory nerves (Kanda, 2013; Langert & Brey, 2018). Perineurial cells and Schwann cells in the peripheral nerves are covered with basement membrane, to which endothelial cells are attached. These basement membranes limit the movement of molecules according to size and electrical charge (Peltonen et al., 2013). The endothelial cells which form the BNB express transporters and receptors to maintain homeostasis in the PNS through the removal of toxic metabolites and incorporation of necessary compounds into the PNS (Sano et al., 2007). The BNB therefore functions as an interface for active material exchange between the endoneurial microenvironment and the blood (Kanda, 2013).

The presence of these barriers can inhibit the delivery of drugs to the peripheral nerve. Administration of analgesics, for example, has been studied in rodent models, showing that the application of hypertonic saline achieves transient opening of the perineurial barrier, allowing drugs to reach their targets (Rittner et al., 2012). In fact, nerve injury provides the opportunity to deliver drugs to desired targets as it may result in the rupture of the perineurial barrier or increase its permeability by disturbing the environment's homeostasis. Additionally, the optimal time to administer the drug can be informed by our understanding of nerve regeneration mechanisms. For example, it has been shown using a rat animal model that (1) within 1 week of injury, a thin perineurial cell layer, and the first evidence of tight junctions are observed, (2) by 3 weeks, this perineurium has formed tight gap junctions. These results have been replicated following ligation of the rat sciatic nerve for 24 h (Hirakawa et al., 2003; Ohta et al., 2005; Peltonen et al., 2013). Conversely, the barriers can act as a protector of nerve allografts at normal permeability levels. This was demonstrated by a nerve allograft animal study, where the animals were not immunocompromised (Zalewski et al., 1993). It was found that long‐term surviving rat nerve allografts are protected by normal permeability barriers provided by the donor perineurium and endoneurial vasculature. Hence, in order to ensure the optimal outcome of a treatment that combines allografts and drugs, the timing of their administration needs to be matched with the ideal nerve regeneration stage.

### 2.2 PNI and treatment

Following PNI, damaged axons and their associated myelin sheaths degenerate distal to the injury site, initiating a cascade of degeneration and repair events. Debris from axon and myelin degradation is cleared, neurons upregulate regeneration associated genes, and distal Schwann cells adopt a growth‐permissive state, guiding new axon sprouts which extend at around 1 mm per day (Burnett & Zager, 2004; K. M. Chan et al., 2014; Fernandez et al., 2018; Standring, 2020). This slow regeneration rate can lead to permanent atrophy and loss of function in muscles which are denervated for prolonged periods (Gulati & Cole, 1990; Isaacs, 2013). Drug treatments are under investigation which can accelerate regeneration after PNI, and as such improve functional recovery. A number of pathways have been identified which play a role in nerve regeneration, for which numerous potential treatment options exist including small molecules and growth factors [reviewed in (Bota & Fodor, 2019; Peltonen et al., 2013)]; however, to date, there are no drug therapies established for clinical use to improve regeneration after nerve injury.

Treatment options for PNI focus on repairing damaged nerves to provide the tissue continuity required to support regeneration. This may be maintained in some injuries where surrounding connective tissue remains intact despite axon damage (neurapraxia, axonotmesis), but in more severe injuries where the nerve is transected (neurotmesis), surgery is used to reconnect the nerve stumps, either directly or using an autograft. Ongoing research aims to develop effective alternatives to the autograft, including allografts, decellularised tissues, and artificial nerve conduits or tissue engineered constructs (Taylor & Haycock, 2020; Wilcox et al., 2020). In all these cases, delivery of therapeutics which increase the rate of regeneration could improve recovery from nerve injuries. In addition, the increasing body of research into the development of allogeneic nerve grafts, including allografts and cellular tissue engineered constructs, may necessitate the need for immunosuppressive treatment. However, Treatments under investigation have not yet been successfully translated to the clinic, in part due to the requirement for improved delivery methods to reduce issues with bioavailability and off target effects, for example through the use of local delivery systems (Rayner, Grillo, et al., 2020).

## 3 LOCAL DRUG DELIVERY

Drugs administered systemically are transported throughout the body, making it challenging to control the dosing profile at a specific target tissue and adverse effects within nontarget organs are common. This has motivated the development of technologies for local drug delivery, one of the most promising of which is biomaterial‐mediated delivery (Fenton et al., 2018). A number of biomaterial‐based drug delivery systems have been clinically approved, including nanoparticles and microparticles, transdermal materials, oral and pulmonary approaches, and implants [reviewed by (Fenton et al., 2018)].

Anesthetics or analgesics are frequently applied to peripheral nerves for the relief of neuropathic pain or to produce regional anesthesia, demonstrating the potential of a local delivery approach for peripheral nerves (Folino & Mahboobi, 2022). In these cases, the nerve is intact, requiring diffusion of compounds through tissue layers and across tissue barriers to enable delivery of relevant doses for effective treatment. This is also true in the case of nerve grafts; for example, rat nerve grafts have been shown to retain or rapidly re‐establish normal permeability barriers (Zalewski et al., 1993) so diffusion across intact barriers into the peripheral nerves may also be required for the delivery of drugs to regenerating nerves after grafting. Regardless of whether we consider intact nerves or nerve grafts, successful administration of therapeutics depends on compound diffusion through nerve tissues, as well as compound solubility in the underlying solute (Langert & Brey, 2018). Numerous studies have sought to characterize these transport parameters and provide a quantitative map of drug movement across different tissue layers in peripheral nerves. The distal nerve trunks have been shown to be relatively impermeable to hydrophilic small molecules such as fluorescein (Abram et al., 2006) and sucrose (Rechthand et al., 1988) as well as large molecules (Poduslo et al., 1994), due to limited intercellular permeability (Liu et al., 2018). However, unlimited passage into the dorsal root ganglion (DRG) has been observed for small and large molecule tracers, attributed to a lack of tight junctions in the microvessel endothelia in the DRG (Abram et al., 2006; Liu et al., 2018).

As discussed, treatments for PNI frequently involve surgical repair; this provides an opportunity for placement of a nerve conduit, graft, or implant which could be used to deliver therapeutics locally. The potential for local delivery of drugs adjacent to the injury site to improve regeneration has been demonstrated with the use of osmotic minipumps. These can provide continuous delivery of drug, and positive functional recovery has been demonstrated in rodent models of PNI following local delivery of the small molecules FK506 (Goldani et al., 2017) and ibuprofen (Rayner et al., 2018). However, minipumps are limited by their size, potential to move from the site of implantation and the need for removal, and numerous other local delivery technologies have been explored. For example, a fibrin gel drug reservoir technique has been developed for delivery of FK506 incorporated in particulate form or PLGA microspheres (Tajdaran et al., 2015). Following transection and repair of rat tibial nerves, this approach was shown to increase the number of myelinated axons detected compared to the control group (Tajdaran et al., 2019). In Labroo et al. (2019), developed a device consisting of two concentric polytetrafluoroethylene tubes with a drug reservoir between them, with diffusion into the lumen controlled by holes in the inner conduit. The device could either bridge the nerve gaps or be placed around a nerve repair or autograft to locally release agents to enhance nerve regeneration (Labroo et al., 2016). It was tested by implantation in a mouse sciatic nerve transection model with a 10 mm nerve gap for delivery of the small molecule FK506 or the growth factor glial cell‐line derived neurotrophic factor (GDNF). A reduction in muscle atrophy, increase in motor endplate reinnervation, increase in compound muscle action potential recovery, and increase in number of myelinated axons was observed in both treatment groups compared to those receiving devices containing media only, showing support for local delivery of these treatments (Labroo et al., 2019). Controlled release of ibuprofen and sulindac sulphide (both peroxisome proliferator‐activated receptor gamma agonists) has been tested using of various drug‐loaded polymers. In these studies, increased axon counts were detected in the distal stump of injured rat nerves following local delivery of ibuprofen from ethylene vinyl acetate (EVA) tubes and electrospun PLGA/ibuprofen or sulindac sulfide nanofibers formed into a nerve wrap (Rayner, Healy, & Phillips, 2020).

These data (and others) collectively demonstrate the potential for local delivery of therapeutics to improve regeneration following a PNI (Daeschler et al., 2021; Rayner, Healy, & Phillips, 2020; Tajdaran et al., 2015, 2019; Zuo et al., 2020). However, there remain outstanding issues and limited data on exactly how much drug reaches the target or how to optimize dosing strategies in vivo. Although key parameters such as bioactivity, optimal target dose, and local drug concentration can be quantified in vitro, extrapolating from in vitro to in vivo models and assessing these parameters directly in vivo present a challenge that is not routinely addressed. For example, in vitro models can allow an effective dose to be determined by applying a compound directly to a target cell population such as Schwann cells or neurons. There are numerous examples of this scenario, including neurite outgrowth assays using rodent and human neuronal cell lines, or rat DRG cells which have been used to determine optimal doses of ibuprofen and FK506 (Rayner et al., 2018; Tajdaran et al., 2015). Given the clear limitations of single cell populations in mimicking in vivo physiology, Rayner et al., (2018) explored ibuprofen dosing used a more complex 3D co‐culture system, engineered neural tissue, and demonstrated that this more accurately predicted in vivo performance. This highlights the importance of considering cell‐to‐cell interactions and tissue architecture when optimizing drug therapies, as well as the influence of the extracellular matrix, all of which tend to be omitted in standard in vitro screens and contribute to limitations in predicting in vivo outcomes from in vitro models. This is compounded by difficulty of measurement in vivo, where effects are measured indirectly via histology and functional testing, often weeks after treatment. A further challenge lies in understanding and recapitulating how changes in peripheral neuroanatomy after injury influence delivery of therapeutic compounds to drug targets in the peripheral nerves. For example, after injury the blood‐nerve‐barrier (BNB) can be breached transiently before being restored (Zalewski et al., 1993), allowing ingress of molecules which would normally be excluded (Langert & Brey, 2018; Madduri & Gander, 2012). In order to be able to predict local drug dynamics and therapeutic efficacy based on data from in vitro and in vivo models, it will be important to be able to understand the impact of changes in tissue structure and permeability following nerve damage and repair.

Integrating mathematical models with experimental techniques provides an opportunity to understand better the mechanisms that underpin drug delivery and distribution, and to use this quantitative understanding to inform and test dosing strategies. Such in silico models take as inputs the architecture and characteristics of native nerve, injured nerve, or artificial nerve constructs, along with the physico‐chemical properties of the therapeutic agent(s) and delivery mechanisms, to predict the movement and accumulation of that agent within and around the nerve through time. Indeed, such models can incorporate features as diverse as tissue architecture, cell populations and vascularization, as well biomaterial‐mediated delivery properties, molecule size, diffusion and tissue clearance rates. Computational experiments then explore the impact of different doses and timings of delivery on the concentration of drug reaching the target cells. Additional features may also be explored for example variations in drug solubility due to changes in temperature, drug polarity and pH, improving the applicability of predictions to capture in vivo situations and thus improving the translation of results from in vitro to in vivo settings (Casadei et al., 2022). Furthermore, when model predictions are evaluated in vivo, the modeling predictions will inform a reduced set of more highly informative in vivo experiments to optimize drug dosing and efficacy. The approach also provides a tool to hypothesis test the role of different mechanisms in determining drug distribution and efficacy, and to interpolate between in vitro and in vivo data.

## 4 MATHEMATICAL MODELING OF DRUG DELIVERY

Mathematical models for drug transport in the field of tissue engineering have been extensively explored (Lambrechts et al., 2012); however, these techniques have rarely been applied to the peripheral nerves (R. Coy et al., 2020). In this section, approaches to modeling local drug delivery and distribution are presented as well as the opportunity of applying them to inform PNI repair. Next, local drug delivery mechanisms and parameters, and their mathematical descriptions, are presented. Throughout we provide example experimental approaches to define these parameters. Finally, approaches to combine experimental data with mathematical models and inform local delivery strategies are discussed.

### 4.1 A framework for mathematically modeling local drug distribution

Here we discuss a general framework for mathematically modeling the distribution of drug delivered locally to a peripheral nerve. We present the key transport mechanisms and then provide examples of how they can be tailored to specific delivery scenarios, considering individual mechanisms in turn.

A mathematical model must describe the dominant physical and biological mechanisms of the system. Here we consider the distribution of a solute $\text{concentration}\;\left(C\right),$ the flux of which can be decomposed into a diffusive (assumed proportional to the gradient of solute concentration via Fick's law), and convective flow ($\vec{v}$) component. Consumption, production, degradation, and/or solute binding are captured through a reaction term (R). The evolution of solute i in time and space may be described through the partial differential equation (PDE)

(1) $$\frac{\partial C_{i}\left(x,y,z,t\right)}{\partial t}+\nabla .\left[\vec{v}\left(x,y,z,t\right)C_{i}\left(x,y,z,t\right)\right]=\nabla .\left(D_{\mathrm{ij}}\nabla C_{i}\left(x,y,z,t\right)\right)+R_{i}\left(x,y,z,t\right),$$

where i=1…N and j=1…M label the *N* solutes and the *M* solvents respectfully, $D_{\mathrm{ij}}$ is the diffusion coefficient (of soluteiin solventj),∇ is the Laplacian operator, x,y,andz, are the spatial coordinates and t is time. Equation (1) must be solved subject to appropriate boundary and initial conditions. The boundary conditions describe the solute concentration or flux at the boundaries of the system (and interfaces within it), whereas initial conditions describe how the system is initialized.

Drug release and distribution have been thoroughly reviewed in the literature [e.g., Peppas and Narasimhan (2014)] and can be broadly categorized into four main delivery methods: diffusion, chemical, osmotic or swelling/erosion controlled. For PNI repair, flow‐mediated delivery (e.g., via injections) is also relevant and Equation (1) can be adapted to capture each scenario. We focus on literature examples of modeling solute delivery in PNI, including growth factors such as nerve growth factor (NGF) (Rosner et al., 2003; Rutkowski & Heath, 2002) and vascular endothelial growth factor (VEGF) (R. Coy et al., 2020) and, to the authors' best knowledge, the first in silico model of drug delivery (Labroo et al., 2021). These examples span the use of cells, Nano particles, biomaterials, and local drug reservoirs as delivery mechanisms.

**FIGURE 2:**
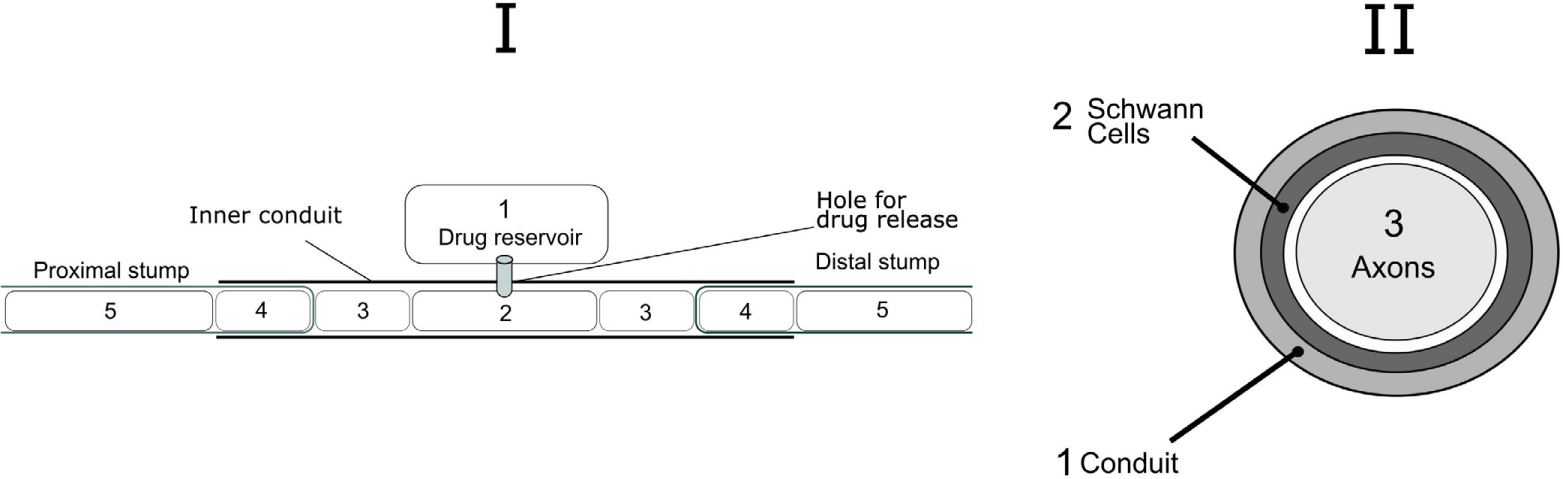
Example geometries for model development. (a) 1D models ‐ different rectangular compartments represent zones with different properties including the drug reservoir (1), drug‐loaded region (2), a section just outside the distal and proximal stumps (3), a region right inside the stumps (4) and a region further inside the stumps (5), [Labroo et al. (2021)]. (b) 2D models—A cross‐section with different annulus zones to capture, for example, tissue made exclusively by axons, surrounding Schwann cells and outer biodegradable material [e.g., Rutkowski and Heath (2002)].

In the examples that follow, we consider two geometrical setups. The first is one dimensional (1D) and captures different regions of the nerve or delivery method using rectangular compartments. Labroo et al. (2021) is one example of this, where a drug‐loaded region, distal and proximal nerve stump compartments have distinct properties (such as diffusion coefficients) (see Figure 2). The second scenario is two‐dimensional and focused on a circular cross‐section through a nerve (see Figure 2). Here the nerve is assumed homogeneous down its length and different zones through the cross‐section are considered. For example, Rutkowski and Heath (2002) consider three concentric annuli to capture (1) a biodegradable material surrounding the nerve, (2) a layer of Schwann cells surround, and (3) axon. Both scenarios simplify the more complex real‐world architecture but provide sufficient structural information to capture the key delivery and transport mechanisms; nonetheless, these geometrical assumptions could be adapted and expanded in line with future research questions.

Next, we consider each of the key transport mechanisms captured by Equation (1) in turn: (a) diffusion (Section 4.2), (b) flow‐driven convection (Section 4.3), (c) reaction (incorporating production, metabolism, binding, decay, Section 4.5). Finally, we provide examples from the literature as summarized in Table 1, including the experimental setups used to characterize the various transport characteristics.

**TABLE 1:** Summary of different mathematical models of solute transport in PNI repair constructs

Paper reference	Model type	Soluble factors	Nerve repair construct type	Data for parameter assignment
Labroo et al. (2021)	Diffusion	Diffusion of tacrolimus	Empty tube	Diffusion rate calculated from in vitro measurements in the literature.
Rutkowski and Heath (2002)	Diffusion–reaction	Release of NGF from nanoparticles	Collagen based hydrogel with embedded nanoparticles	Release rate from nanoparticles measured in vitro. In vitro diaphragm experiments used to estimate the diffusion coefficient of NGF in collagen‐based hydrogel
Rosner et al. (2003)	Diffusion–reaction	Release of NGF from Schwann cells	Porous wrap	NGF diffusion rate estimated from in vitro diaphragm experiments. Tortuosity of the porous tube measured using in vitro diaphragm experiments. Uptake rate of NGF by neurites evaluated using in silico experiments.
R. Coy et al. (2020)	Diffusion–reaction	Release of VEGF from endothelial cells	Adipose‐derived stem cell‐seeded collagen hydrogels	In vitro experiments at a range of seeded cell densities and ambient oxygen conditions

### 4.2 Solute diffusion

Diffusion is the passive movement of molecules or particles from regions of higher to regions of lower concentration (Jacobs, 1935). It is a key transport mechanism governing how solutes distribute within the water phase of tissues and is included in all mathematical models of therapeutic solute distribution in PNI treatment (including all examples in Table 1). Diffusion is represented by the third term on the right‐hand side of Equation (1) and is characterized by a diffusion coefficient (units m^2^/s), which captures the diffusion of a molecule or particles within a particular substrate. Often diffusion coefficients are estimated based on empirical relationships, for example in terms of the molecule/particles Stokes' radius, temperature, however, it remains rare for such estimates to be calibrated against dedicated measurements.

The examples in Table 1 cover a range of methods for estimating diffusion coefficients for solutes in different fluid‐based materials. In Labroo et al., 2021 the diffusion coefficient for tacrolimus is estimated by extrapolating from measurements made on fluorescently labeled molecules by Popov and Poo (1992). The authors injected fluorescently labeled molecules into the soma of Xenopus neurons for molecules of increasing size and tracked their movement over time to extract an empirical relationship between intracellular diffusion coefficient and molecule size. Labroo et al., 2021 then used the diffusion coefficient for a molecule size equivalent to tacrolimus. Rutkowski and Heath (2002) used a similar approach to estimate the diffusion coefficient of NGF in nerve tissue. Swabb et al. (1974) developed a linear relationship between molecular weight and diffusion coefficient based on measurements of movement of different molecular weight molecules across animal tumor tissue slides in vitro. Rutkowski and Heath (2002) then extracted the corresponding value of the diffusion coefficient for a molecule with the same molecular weight as NGF. In both of these examples, the approach is limited as the substrate (e.g., tissue type) will influence diffusion characteristics of the drug/material studies.

**FIGURE 3:**
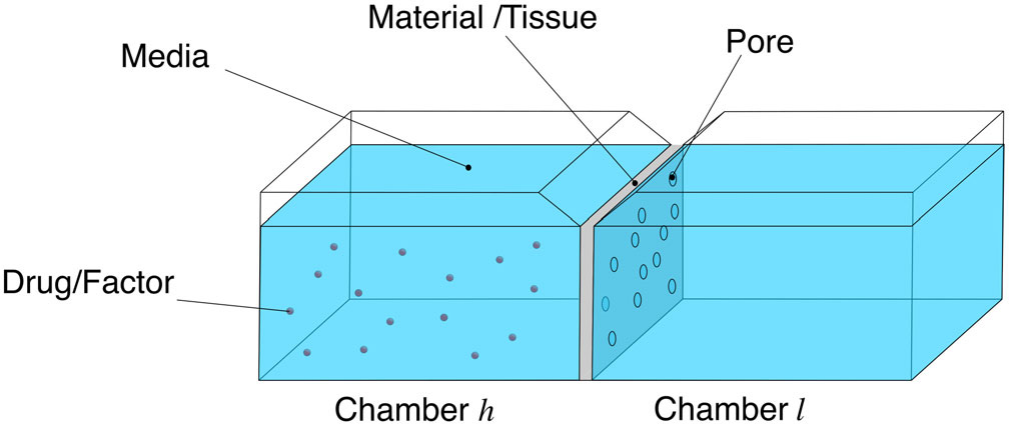
A dual‐chamber setup for measuring the diffusivity of drugs and other soluble factors in hydrogels, developed by Rutkowski and Heath (2002). The drug/factor is introduced into chamber h which is separated from chamber l by a diaphragm fabricated from the material of interest (e.g., hydrogel). The concentration of drug/factor in chamber l is measured in time and the corresponding diffusion coefficient calculated using Equation (2).

To address this, various authors have used an in vitro setup to measure the diffusion of NGF in materials such as collagen‐based hydrogels (Rutkowski & Heath, 2002). The setup consists of two chambers containing the same fluid substrate (e.g., phosphate‐buffered saline), separated by a diaphragm fabricated from the material of interest (e.g., collagen‐based hydrogel), as shown in Figure 3. A known volume of solute (at a known concentration) is placed in one chamber and the concentration in the second chamber measured at regular time intervals. The diffusion coefficient is estimated using the relationship developed by Cussler and Cussler (2009).

(2) $$D=\frac{1}{\mathrm{H\beta t}}\ln\left(\frac{C_{l}\left(0\right)-C_{h}\left(0\right)}{C_{l}\left(t\right)-C_{h}\left(t\right)}\right),$$

where $\beta =\frac{A}{l}\left(\frac{1}{V_{l}}+\frac{1}{V_{h}}\right)$ and H is the partition coefficient. Here A and l are the surface area, length of the diaphragm, $V_{h}$, $V_{l}$ and $C_{h}$, $C_{l}$ are the volume and solute concentration in the higher‐ and lower‐concentration chambers, respectively.

Although these are the gold standard approaches in the literature, there remain limitations in accurately predicting diffusion parameters. It remains rare to evaluate diffusion parameters for specific substrate/solute combinations and as a result, most studies rely on extrapolating data calibrated for different solutes or materials. It is also commonly assumed that the properties of nerve tissue are homogeneous and do not change in time through the regeneration process. With the advent of new technologies that enable quantification of diffusion parameters in space and time (e.g., fluorescence quantification after photobleaching, photoactivation, photoconversion, photo‐switching; Lambrechts et al., 2012), the importance of these assumptions can be assessed and updated.

### 4.3 Flow‐dependent drug delivery

Flow‐dependent (or convective) drug transport in PNI repair may be induced by local delivery methods, for example via minipumps or injections. Further, the hydrogels which form the basis of nerve repair constructs (such as collagen‐based materials) erode and swell inducing local fluid movement. Such flow‐dependent transport effects are captured by the second term in the left‐hand side of Equation (1), where $\vec{v}$ describes the fluid velocity vector.

**FIGURE 4:**
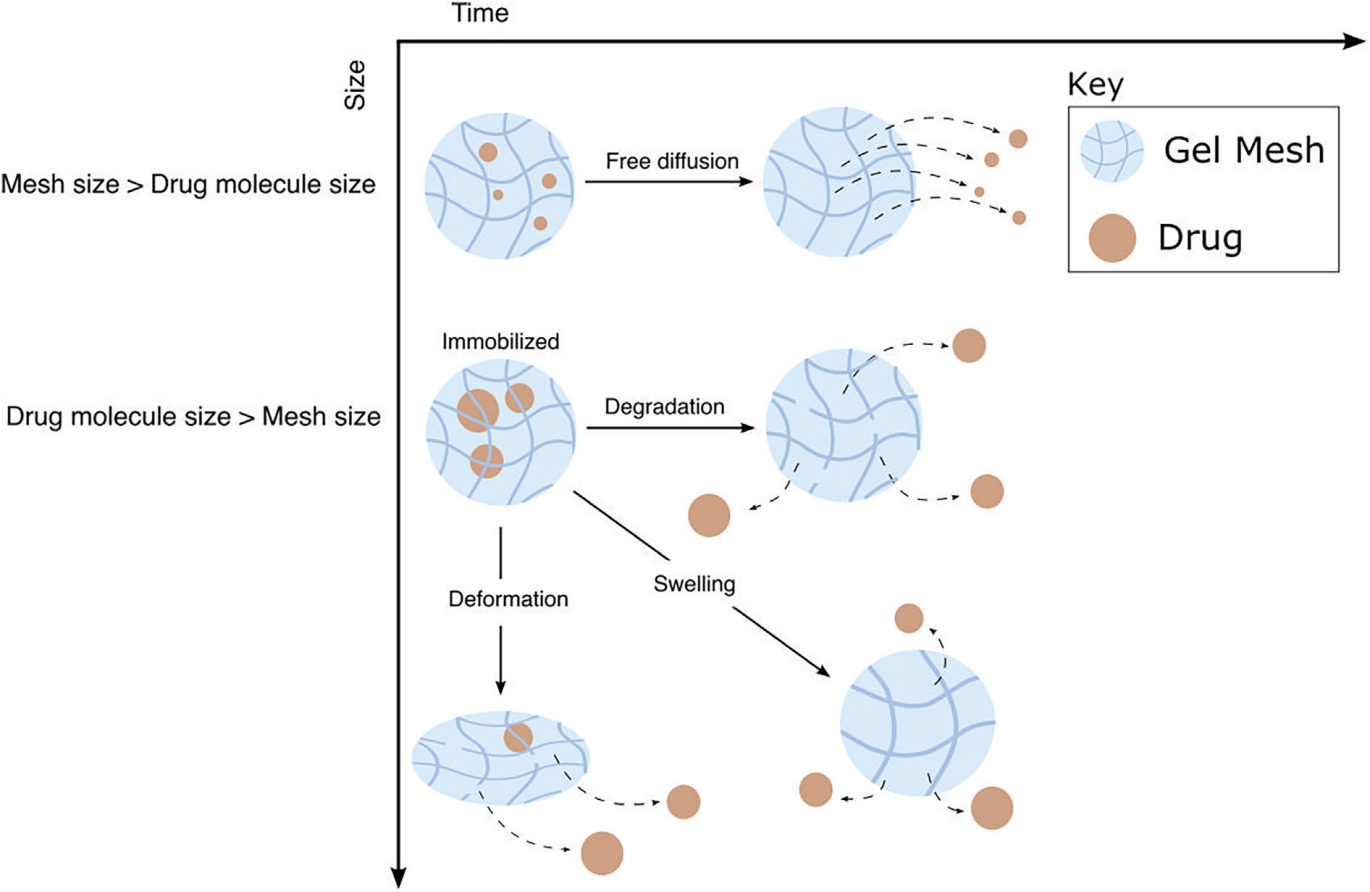
Schematic description, adapted from Li & Mooney, 2016, of the link between hydrogel matrix properties and drug delivery in the context of hydrogel deformation, swelling, and matrix degradation

Hydrogels, which comprise cross‐linked polymer networks, fluid and therapeutic cells, are common substrates for engineered constructs for PNI repair. Various studies have explored the importance of degradation and swelling of such hydrogels for controlling their physical properties and resultant drug release dynamics (see Li and Mooney (2016); Figure 4 for a schematic representation). Mesh size is one such important property which may be controlled via the concentration of polymer/cross‐linker, as well as pH, temperature. If the drug molecule is smaller than the mesh size, the drug will diffuse freely within the hydrogel; indeed, this is assumed across all examples presented in Table 1. If a molecule is smaller than the mesh size, it will be immobilized and instead only released via swelling, deformation, or degradation of the mesh. Numerous mathematical models have sought to capture these dynamics (see Caccavo (2019)). Wu and Brazel (2008) described hydrogel swelling via a volume‐change model, parameterized via dissolution experiments that explored the link between drug release and matrix properties. Caccavo (2019) expanded the approach to quantify the hydrogel deformation as it dissolved using nuclear magnetic resonance (NMR) imaging, and then compared the differing drug release profiles induced by two different hydrogel formulations, as predicted via in silico models.

Although rare in the PNI repair field, mathematical models of slow flow dynamics in other fields could be adapted to describe, for example, the impact of minipumps, injections, and the contribution of microvasculature‐induced flows. Cancer modeling is a ripe field for technology transfer, not only through the development of sophisticated mathematical models of microvascular and extravascular flows (Berg et al., 2022; Dogra et al., 2020; Shipley et al., 2019; Stamatelos et al., 2014; Sweeney et al., 2019), but also by highlighting the importance of experimental and/or imaging data to characterize these flows in situ (d'Esposito et al., 2018). Similarly, mathematical models of fluid flows in tissue engineering and regenerative medicine is evolving to become a rich research field including, for example, describing perfusion of fluid in and around hydrogel‐based constructs (O'Dea et al., 2012; Waters et al., 2021). This remains and exciting and open challenge in local drug delivery, not only in PNI repair.

### 4.4 Production, metabolism, and binding

Finally, the reaction term in Equation (1) $\left(R_{i}\left(x,y,z,t\right)\;\text{for solute}\;i\right)$ can be tailored to describe any source or sink of solute, for example, production, decay, binding, and uptake of soluble factors and drugs. Whilst these have not been explored for drugs for PNI repair, various studies have characterized this reaction term for different soluble factors (such as growth factors) in this setting (e.g., R. Coy et al., 2020; Rosner et al., 2003; Rutkowski & Heath, 2002). We discuss these here, particularly highlighting the role of experimental data in informing the functional forms used for $R_{i}\left(x,y,z,t\right)$.Rosner et al. (2003) and Rutkowski and Heath (2002) both develop models for the release of NGF, the former from microparticles and the latter from Schwann cells. Both implemented in vitro protocols where the cells or microparticles were placed in media and the release rate of NGF measured by measuring the concentration of NGF in media samples over time using ELISA. A functional form (such as a polynomial) was fitted to these data, although these relationships were not linked to any fundamental principles that governed the system behavior. Therefore, although the relationships fitted the in vitro data well, model predictions cannot be extended beyond the regime of data collection.

Rutkowski and Heath (2002) also modeled the uptake of NGF by neurons. Here, instead of directly fitting a parameter/polynomial to data, the underlying understanding of how neurites uptake NGF was considered. NGF (denoted N) needs to bind to two receptors, the low affinity p75^NGFR^ (denoted L) and the high affinity trkA (denoted H), before it is taken up. The uptake kinetics can, therefore, be described by

(3) $$\begin{array}{ccc} N+H\overset{K_{H}}{↔}\mathrm{NH}+L & \overset{K_{\mathrm{HL}}}{\to } & \mathrm{NHL}\\ N+L\overset{K_{L}}{↔}\mathrm{NL}+H & \overset{K_{\mathrm{LH}}}{\to } & \mathrm{NHL} \end{array}$$

where $K_{H}$, $K_{L}$ represent the binding rates to form the intermediate compounds, and $K_{\mathrm{HL}}$, $K_{\mathrm{LH}}$ the rates associated with the formation of the final product. To parameterize these rates, co‐cultures of dorsal root ganglia neurons and Schwann cells from Sprague Dawley rats were performed. The cultures were incubated with a known concentration of NGF for 5 days and the NGF concentration in media samples measured at different time points. A curve describing the NGF uptake by neurons over time was then used to infer the binding rates in Equation (3). Therefore, the biochemistry of the system was used to inform these binding rates via the in vitro data. A further extension might explore how these rates depend on other key features such as the number of neurons, concentration of NGF, so that the resulting model could be applied more broadly.

Finally, Coy et al. (2020) sought to optimize the seeded density of therapeutic cells (adipose‐derived Schwann cells) for PNI repair in engineered constructs. In particular, they explored the role of a low‐oxygen environment in modulating production of VEGF by the therapeutic cell population, and how this depends on the cell density. The study included a dedicated set of in vitro experiments where cells embedded in collagen hydrogels at five different seeded densities were cultured at five different ambient oxygen conditions. VEGF concentration in the surrounding media was measured at 24 h for all conditions. A functional relationship for the production rate of VEGF as a function of oxygen concentration was established using a combination of understanding existing models available in the literature as well as trends in the measured data (e.g., evidence for a threshold oxygen concentration at which VEGF is upregulated),

(4) $$R\left(n,c\right)=\mathrm{\alpha n}\left(\frac{\left(V_{m}+1\right)}{2}-\frac{\left(V_{m}-1\right)}{2}\tanh\left(B_{v}\left(c-c_{h}\right)\right)\right)$$

where n is the cell density, c the oxygen concentration, α is the baseline production of VEGF, $c_{h}$ is the hypoxia threshold at which VEGF production is upregulated, $B_{v}$ is the gradient of the oxygen‐dependent upregulation and $V_{m}$ is a VEGF upregulation factor. All these parameters were fitted to the data, as in the other two cases. However, here, the model was derived from the literature and the data captures nonlinearities between the variables simulated. This means the model can be more readily extrapolated to new scenarios.

These three examples capture the most common approaches in the literature to model different aspects of PNI repair (although not local drug delivery). Across these examples, it is clear that underpinning model development by measurements is essential to ensure that the models are predictive. In the next section, we explore this in more detail and propose how a combined approach of experimentation and modeling could be used to develop local drug delivery strategies for PNI repair.

### 4.5 Making model predictions to inform local drug delivery protocols

The previous sections describe how mathematical models of drug delivery and distribution may be setup, including how parameters within the models can be identified through dedicated experiments. Once this is complete, models may be used to make predictions, for example on how a particular drug distributes in space and time for a given delivery modality, or how to optimize a dosing profile to achieve a particular outcome. Here we describe the approaches taken by the studies summarized in Table 1 and discuss how a combined mathematical‐experimental approach may be taken forward to inform local drug delivery technologies.

Rutkowski and Heath (2002) sought to identify the size and number of hydrogel‐embedded nanoparticles that will deliver a desired dose of NGF. To achieve this, they performed 100s of simulations where those two parameters are changed within a chosen range (ensuring that the parameter space is covered homogenously) to test the impact on NGF delivery and thus identified the best parameter combinations to achieve a particular NGF profile. Rosner et al. (2003) expanded the approach to test the impact of hydrogel porosity and thickness on the release profile of NGF by Schwann cells, and Labroo et al. (2021), similarly, tested the impact of the porosity of the drug delivery compartment on the release profile of TAK. These models demonstrate that a mathematical model can be developed based on routinely collected data, with established physical principles. However, the limitations in the model predictions will echo the limitations of the in vitro model employed to parameterize the model. For example Rutkowski and Heath (2002) ignores that Schwann cells also have receptors for NGF (J. R. Chan et al., 2004), and this presents challenges for validating the model predictions.

**FIGURE 5:**
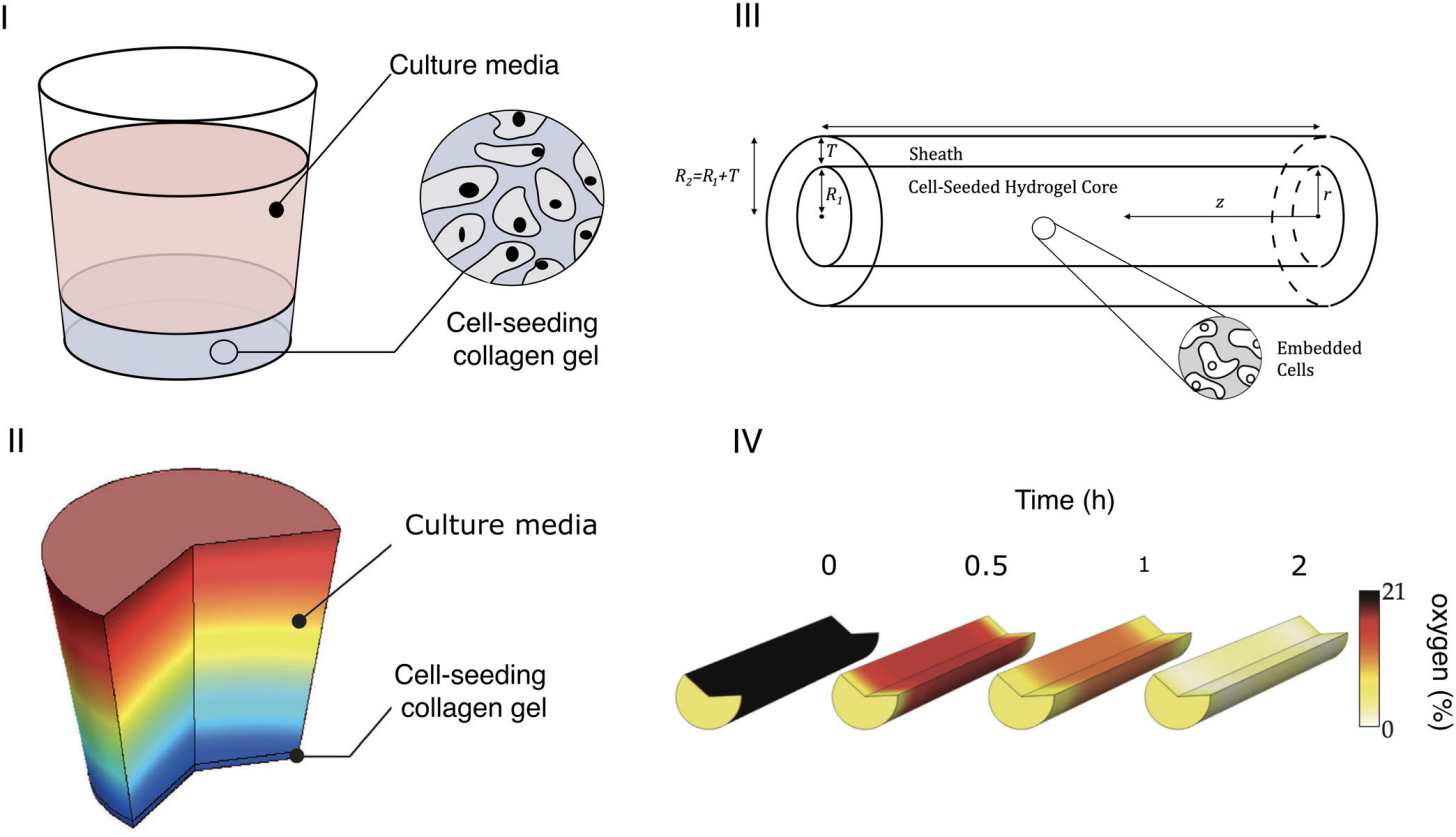
Overview of the workflow followed by the work by Coy and collaborators. Firstly an in vitro setup is devised (a) adapted from R. H. Coy, 2020, where oxygen, cell‐seeding density and VEGF can be monitored. The data then inform a mathematical model as demonstrated in (b), which presents the initial oxygen distribution (10% at the top, 9.6% at the bottom), for a simulation adapted from R. Coy et al., 2020. The model can then optimize for cell viability based on all the variables simulated. The optimum conditions can then be extrapolated to a cylindrical geometry (c) adapted from R. H. Coy, 2020 and the variables optimized for this geometry. In (d) adapted from R. H. Coy, 2020 simulations of oxygen concentration over time are presented.

Coy et al. (2020) aims to overcome the limitations found in the models in Table 1 by developing an in vitro setup that accounts for a larger number of variables and the relationship between them (cell number, oxygen and VEGF concentrations). The complexity of the in vitro setup enabled model parameters to be informed, similar to those of the earlier works (diffusivities, hydrogel properties, etc) as well as how these parameters impact the distribution of key factors within their in vitro test setup (Figure 5a,b). The model was then applied to a cylindrical geometry representative of a nerve repair construct (i.e., in vivo testing scenario, Figure 5c). Again, key parameters were varied to explore the impact on predictions of the oxygen (Figure 5d), cell and VEGF distributions including the seeded cell density and oxygen pre‐culture conditions that promote cell survival in vivo.

In each example, a large number of simulations are performed to provide the required insights. This may come at high computational cost and the outcomes depend on the robustness of the original model parameterization. However, these limitations are offset by the opportunity to provide meaningful data and insights that would be highly challenging (and costly) to achieve using experiments in isolation, in broader alignment with important goals to reduce, refine and replace animal experimentation (Lemon, 2018). Numerous other mathematical approaches exist for optimizing model predictions that more efficiently explore the parameter space. These include genetic algorithms utilizing the rules that govern genetic evolution and have proven effective in a variety of related fields (Laranjeira et al., 2022; Mirchi & Soltani, 2020).

## 5 CONCLUSION

**FIGURE 6:**
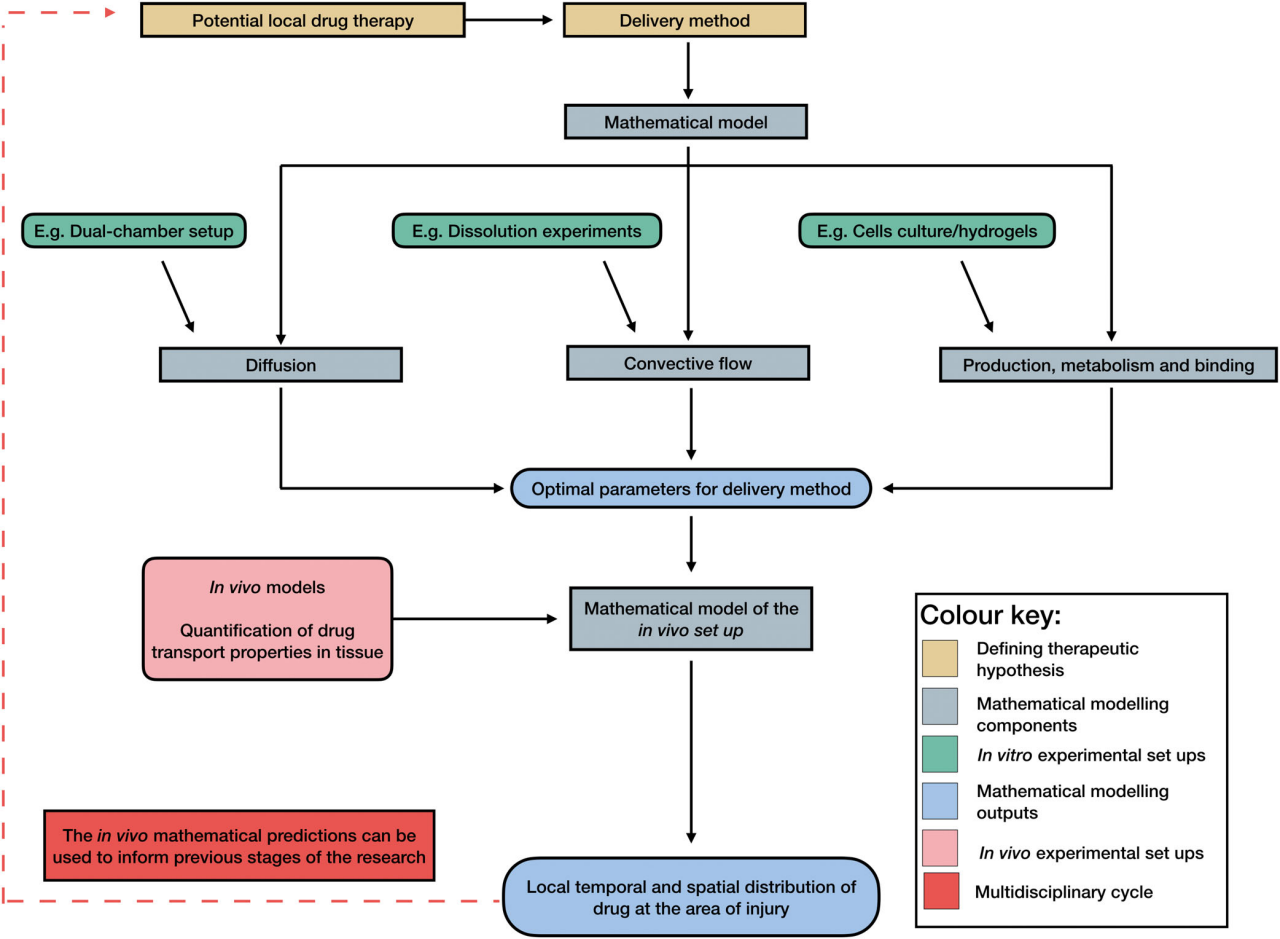
Diagram of multidisciplinary workflow, where the interaction of mathematical modeling and experimental set up are clearly discriminated

We conclude by proposing a combined approach of modeling and experimentation to accelerate the development of local drug delivery technologies for PNI repair (see Figure 6). First, a drug and the method delivery are proposed. A mathematical model is put forward, encapsulating the key transport features (diffusion, flow‐mediated delivery, appropriate uptake, binding etc. kinetics). As discussed in Section 4, key parameters and relationships in these models may be determined via carefully‐designed, dedicated in vitro experiments (e.g., to characterize the release rate of drug from biomaterials, the metabolism/uptake of the drug by relevant cell populations, the diffusivity of a drug in different tissue layers, or the fluid flows induced via an injection). Once these in vitro experiments are complete, parameters in the model may be identified providing a quantitative tool to explore different delivery regimes in vitro. This tool can be used to perform virtual simulations of different delivery scenarios in vitro, and to test the robustness of model predictions against available data.

Next, the validated model may be applied to geometries and delivery scenarios representative of an in vivo (e.g., animal model) scenario. Whilst there are abundant assumptions associated with extrapolating from in vitro to in vivo setups, this approach provides additional information to inform and refine the set of in vivo experiments to those that will provide the most valuable information. This includes generating predictions of drug distributions (and resultant impact on cells) throughout space and time—data that are not available using experimental methods in isolation. For example, the approach may inform a narrower range of drug concentrations encapsulated in a biomaterial, with a higher potential for efficacy in terms of improving nerve regeneration. Model predictions and parameter regimes should be tested against (in vivo) experimental measurements at individual time points and spatial locations and this may inform further updates in the mathematical‐experimental framework. Finally, the validated framework may be used to refine the drug delivery approach, thus accelerating the translation of drug delivery strategies for treating PNI repair.

## AUTHOR CONTRIBUTIONS

**Simao Laranjeira:** Conceptualization (equal); investigation (equal); methodology (equal); writing – original draft (equal); writing – review and editing (equal). **Victoria H. Roberton:** Conceptualization (equal); investigation (equal); methodology (equal); writing – original draft (equal); writing – review and editing (equal). **James B. Phillips:** Funding acquisition (equal); project administration (equal); supervision (equal); writing – review and editing (equal). **Rebecca J. Shipley:** Funding acquisition (equal); project administration (equal); supervision (equal); writing – review and editing (equal).

## FUNDING INFORMATION

This work was supported by funding from the EPSRC (EP/R004463/1) and the 2018 UCL Rosetrees Stoneygate Prize.

## CONFLICT OF INTEREST

The authors have declared no conflicts of interest for this article.

## RELATED WIREs ARTICLE

Axon repair: Surgical application at a subcellular scale

## Data Availability

Data sharing is not applicable to this article as no new data were created or analyzed in this study.

## References

[wsbm1593-bib-0001] Abram, S. E. , Yi, J. , Fuchs, A. , & Hogan, Q. H. (2006). Permeability of injured and intact peripheral nerves and dorsal root ganglia. Anesthesiology, 105(1), 146–153. 10.1097/00000542-200607000-00024 16810006

[wsbm1593-bib-0002] Allt, G. , & Lawrenson, J. G. (2000). The blood‐nerve barrier: Enzymes, transporters and receptors—A comparison with the blood‐brain barrier. Brain Research Bulletin, 52(1), 1–12. 10.1016/s0361-9230(00)00230-6 10779695

[wsbm1593-bib-0003] Azzam, N. A. , Zalewski, A. A. , Williams, L. R. , & Azzam, R. N. (1991). Nerve cables formed in silicone chambers reconstitute a perineurial but not a vascular endoneurial permeability barrier. The Journal of Comparative Neurology, 314(4), 807–819. 10.1002/cne.903140413 1816277

[wsbm1593-bib-0004] Berg, M. , Holroyd, N. , Walsh, C. , West, H. , Walker‐Samuel, S. , & Shipley, R. (2022). Challenges and opportunities of integrating imaging and mathematical modelling to interrogate biological processes. The International Journal of Biochemistry & Cell Biology, 146, 106195. 10.1016/j.biocel.2022.106195 35339913 PMC9693675

[wsbm1593-bib-0005] Bota, O. , & Fodor, L. (2019). The influence of drugs on peripheral nerve regeneration. Drug Metabolism Reviews, 51(3), 266–292. 10.1080/03602532.2019.1632885 31203666

[wsbm1593-bib-0006] Burnett, M. G. , & Zager, E. L. (2004). Pathophysiology of peripheral nerve injury: A brief review. Neurosurgical Focus, 16(5), 1–7. 10.3171/foc.2004.16.5.2 15174821

[wsbm1593-bib-0007] Caccavo, D. (2019). An overview on the mathematical modeling of hydrogels' behavior for drug delivery systems. International Journal of Pharmaceutics, 560, 175–190. 10.1016/j.ijpharm.2019.01.076 30763681

[wsbm1593-bib-0008] Casadei, B. , Giacosa, M. , Maula, A. , Plos, S. , Zappulla, L. , Viotto, C. , Deriu, M. A. , & Tuszynski, J. A. (2022). Complexities of drug resistance in cancer: An overview of strategies and mathematical models. In I. Balaz & A. Adamatzky (Eds.), Cancer, complexity, computation (Vol. 46, pp. 309–332). Springer International Publishing. 10.1007/978-3-031-04379-6_14

[wsbm1593-bib-0009] Chan, J. R. , Watkins, T. A. , Cosgaya, J. M. , Zhang, C. , Chen, L. , Reichardt, L. F. , Shooter, E. M. , & Barres, B. A. (2004). NGF controls axonal receptivity to myelination by Schwann cells or oligodendrocytes. Neuron, 43(2), 183–191. 10.1016/j.neuron.2004.06.024 15260955 PMC2758239

[wsbm1593-bib-0010] Chan, K. M. , Gordon, T. , Zochodne, D. W. , & Power, H. A. (2014). Improving peripheral nerve regeneration: From molecular mechanisms to potential therapeutic targets. Experimental Neurology, 261, 826–835. 10.1016/j.expneurol.2014.09.006 25220611

[wsbm1593-bib-0011] Coy, R. , Al‐Badri, G. , Kayal, C. , O'Rourke, C. , Kingham, P. J. , Phillips, J. B. , & Shipley, R. J. (2020). Combining *in silico* and *in vitro* models to inform cell seeding strategies in tissue engineering. Journal of the Royal Society Interface, 17(164), 20190801. 10.1098/rsif.2019.0801 32208821 PMC7115239

[wsbm1593-bib-0012] Coy, R. H. (2020). Modelling the impact of cell seeding strategies on cell survival and vascularisation in engineered tissue [PhD thesis]. UCL (University College London).

[wsbm1593-bib-0013] Cussler, E. L. , & Cussler, E. L. (2009). Diffusion: Mass transfer in fluid systems. Cambridge University Press.

[wsbm1593-bib-0014] Daeschler, S. C. , Chan, K. , Feinberg, K. , Manoraj, M. , Cheung, J. , Zhang, J. , Mirmoeini, K. , Santerre, J. P. , Gordon, T. , & Borschel, G. H. (2021). A biodegradable, tacrolimus‐releasing nerve wrap promotes peripheral nerve regeneration. bioRxiv. 10.4103/NRR.NRR-D-22-01198 PMC11246136 38767493

[wsbm1593-bib-0015] d'Esposito, A. , Sweeney, P. W. , Ali, M. , Saleh, M. , Ramasawmy, R. , Roberts, T. A. , Agliardi, G. , Desjardins, A. , Lythgoe, M. F. , Pedley, R. B. , Shipley, R. , & Walker‐Samuel, S. (2018). Computational fluid dynamics with imaging of cleared tissue and of in vivo perfusion predicts drug uptake and treatment responses in tumours. Nature Biomedical Engineering, 2(10), 773–787. 10.1038/s41551-018-0306-y 31015649

[wsbm1593-bib-0016] Dogra, P. , Butner, J. D. , Ruiz Ramírez, J. , Chuang, Y. , Noureddine, A. , Jeffrey Brinker, C. , Cristini, V. , & Wang, Z. (2020). A mathematical model to predict nanomedicine pharmacokinetics and tumor delivery. Computational and Structural Biotechnology Journal, 18, 518–531. 10.1016/j.csbj.2020.02.014 32206211 PMC7078505

[wsbm1593-bib-0017] Fenton, O. S. , Olafson, K. N. , Pillai, P. S. , Mitchell, M. J. , & Langer, R. (2018). Advances in biomaterials for drug delivery. Advanced Materials, 30(29), 1705328. 10.1002/adma.201705328 PMC6261797 29736981

[wsbm1593-bib-0018] Fernandez, L. , Komatsu, D. E. , Gurevich, M. , & Hurst, L. C. (2018). Emerging strategies on adjuvant therapies for nerve recovery. The Journal of Hand Surgery, 43(4), 368–373. 10.1016/j.jhsa.2018.01.023 29618417

[wsbm1593-bib-0019] Folino, T. B. , & Mahboobi, S. K. (2022). Regional anesthetic blocks. StatPearls Publishing Retrieved from http://www.ncbi.nlm.nih.gov/books/NBK563238/ 33085385

[wsbm1593-bib-0020] Goldani, E. , Antônio, M. , Gelain, S. , Cardoso, T. M. , Pellizzari, A. C. , Curra, M. D. , Mariel, T. , Beuren, A. , Rangel, J. O. , & Silva, J. B. (2017). Locally applied FK506 improves functional recovery in rats after sciatic nerve transection. International Journal of Innovative Research Medical Science, 2(6), 789–796.

[wsbm1593-bib-0021] Gulati, A. K. , & Cole, G. P. (1990). Nerve graft immunogenicity as a factor determining axonal regeneration in the rat. Journal of Neurosurgery, 72(1), 114–122. 10.3171/jns.1990.72.1.0114 2294170

[wsbm1593-bib-0022] Hirakawa, H. , Okajima, S. , Nagaoka, T. , Takamatsu, T. , & Oyamada, M. (2003). Loss and recovery of the blood‐nerve barrier in the rat sciatic nerve after crush injury are associated with expression of intercellular junctional proteins. Experimental Cell Research, 284(2), 194–208. 10.1016/S0014-4827(02)00035-6 12651153

[wsbm1593-bib-0023] Hirasawa, Y. , Saiki, T. , Nakao, Y. , & Katsumi, Y. (1994). Regeneration of perineurium after nerve injury and autografting. International Orthopaedics, 18(4), 229–235. 10.1007/BF00188327 8002112

[wsbm1593-bib-0024] Isaacs, J. (2013). Major peripheral nerve injuries. Hand Clinics, 29(3), 371–382. 10.1016/j.hcl.2013.04.006 23895717

[wsbm1593-bib-0025] Jacobs, M. H. (1935). Diffusion processes. In M. H. Jacobs (Ed.), Diffusion processes (pp. 1–145). Springer. 10.1007/978-3-642-86414-8_1

[wsbm1593-bib-0026] Kanda, T. (2013). Biology of the blood–nerve barrier and its alteration in immune mediated neuropathies. Journal of Neurology, Neurosurgery, and Psychiatry, 84(2), 208–212. 10.1136/jnnp-2012-302312 23243216

[wsbm1593-bib-0027] Kristensson, K. , & Olsson, Y. (1971). The perineurium as a diffusion barrier to protein tracers. Acta Neuropathologica, 17(2), 127–138. 10.1007/BF00687488 5101596

[wsbm1593-bib-0028] Labroo, P. , Hilgart, D. , Davis, B. , Lambert, C. , Sant, H. , Gale, B. , Shea, J. E. , & Agarwal, J. (2019). Drug‐delivering nerve conduit improves regeneration in a critical‐sized gap. Biotechnology and Bioengineering, 116(1), 143–154. 10.1002/bit.26837 30229866

[wsbm1593-bib-0029] Labroo, P. , Ho, S. , Sant, H. , Shea, J. , Gale, B. K. , & Agarwal, J. (2016). Controlled delivery of FK506 to improve nerve regeneration. Shock, 46(3) suppl 1, 154–159. 10.1097/SHK.0000000000000628 27058050

[wsbm1593-bib-0030] Labroo, P. , Ho, S. , Sant, H. , Shea, J. E. , Agarwal, J. , & Gale, B. (2021). Modeling diffusion‐based drug release inside a nerve conduit in vitro and in vivo validation study. Drug Delivery and Translational Research, 11(1), 154–168. 10.1007/s13346-020-00755-y 32367424

[wsbm1593-bib-0031] Lambrechts, D. , Schrooten, J. , Van de Putte, T. , & Van Oosterwyck, H. (2012). Computational modeling of mass transport and its relation to cell behavior in tissue engineering constructs. In L. Geris (Ed.), Computational modeling in tissue engineering (Vol. 10, pp. 85–105). Springer. 10.1007/8415_2012_139

[wsbm1593-bib-0032] Langert, K. A. , & Brey, E. M. (2018). Strategies for targeted delivery to the peripheral nerve. Frontiers in Neuroscience, 12, 887. 10.3389/fnins.2018.00887 30542262 PMC6277764

[wsbm1593-bib-0033] Laranjeira, S. , Pellegrino, G. , Bhangra, K. S. , Phillips, J. B. , & Shipley, R. J. (2022). *In silico* framework to inform the design of repair constructs for peripheral nerve injury repair. Journal of the Royal Society Interface, 19(188), 20210824. 10.1098/rsif.2021.0824 35232275 PMC8889181

[wsbm1593-bib-0034] Lemon, R. N. (2018). Applying the 3Rs to neuroscience research involving nonhuman primates. Drug Discovery Today, 23(9), 1574–1577. 10.1016/j.drudis.2018.05.002 29733893

[wsbm1593-bib-0035] Li, J. , & Mooney, D. J. (2016). Designing hydrogels for controlled drug delivery. Nature Reviews Materials, 1(12), 16071. 10.1038/natrevmats.2016.71 PMC5898614 29657852

[wsbm1593-bib-0036] Liu, H. , Chen, Y. , Huang, L. , Sun, X. , Fu, T. , Wu, S. , Zhu, X. , Zhen, W. , Liu, J. , Lu, G. , Cai, W. , Yang, T. , Zhang, W. , Yu, X. , Wan, Z. , Wang, J. , Summerfield, S. G. , Dong, K. , & Terstappen, G. C. (2018). Drug distribution into peripheral nerve. Journal of Pharmacology and Experimental Therapeutics, 365(2), 336–345. 10.1124/jpet.117.245613 29511033

[wsbm1593-bib-0037] Lundborg, G. (1988). Intraneural Microcirculation. Orthopedic Clinics of North America, 19(1), 1–12. 10.1016/S0030-5898(20)30326-6 3275919

[wsbm1593-bib-0038] Madduri, S. , & Gander, B. (2012). Growth factor delivery systems and repair strategies for damaged peripheral nerves. Journal of Controlled Release, 161(2), 274–282. 10.1016/j.jconrel.2011.11.036 22178593

[wsbm1593-bib-0039] Mirchi, P. , & Soltani, M. (2020). Estimation of drug and tumor properties using novel hybrid meta‐heuristic methods. Journal of Theoretical Biology, 488, 110121. 10.1016/j.jtbi.2019.110121 31857083

[wsbm1593-bib-0040] O'Dea, R. , Byrne, H. , & Waters, S. (2012). Continuum modelling of In vitro tissue engineering: A review. In L. Geris (Ed.), Computational modeling in tissue engineering (Vol. 10, pp. 229–266). Springer. 10.1007/8415_2012_140

[wsbm1593-bib-0041] Ohta, M. , Okajima, S. , Hirakawa, H. , Tokunaga, D. , Fujiwara, H. , Oda, R. , Kobashi, H. , Hirata, M. , & Kubo, T. (2005). Expression of tight and gap junctional proteins in the perineurial window model of the rat sciatic nerve. The International Journal of Neuroscience, 115(10), 1469–1481. 10.1080/00207450591001871 16162451

[wsbm1593-bib-0042] Palispis, W. A. , & Gupta, R. (2017). Surgical repair in humans after traumatic nerve injury provides limited functional neural regeneration in adults. In Experimental neurology (Vol. 290, pp. 106–114). Academic Press. 10.1016/j.expneurol.2017.01.009 28111229

[wsbm1593-bib-0043] Peltonen, S. , Alanne, M. , & Peltonen, J. (2013). Barriers of the peripheral nerve. Tissue Barriers, 1(3), e24956. 10.4161/tisb.24956 24665400 PMC3867511

[wsbm1593-bib-0044] Peppas, N. A. , & Narasimhan, B. (2014). Mathematical models in drug delivery: How modeling has shaped the way we design new drug delivery systems. Journal of Controlled Release, 190, 75–81. 10.1016/j.jconrel.2014.06.041 24998939

[wsbm1593-bib-0045] Poduslo, J. F. , Curran, G. L. , & Berg, C. T. (1994). Macromolecular permeability across the blood‐nerve and blood‐brain barriers. Proceedings of the National Academy of Sciences of the United States of America, 91(12), 5705–5709. 10.1073/pnas.91.12.5705 8202551 PMC44065

[wsbm1593-bib-0046] Popov, S. , & Poo, M. (1992). Diffusional transport of macromolecules in developing nerve processes. The Journal of Neuroscience, 12(1), 77–85. 10.1523/JNEUROSCI.12-01-00077.1992 1370324 PMC6575694

[wsbm1593-bib-0047] Rayner, M. L. D. , Grillo, A. , Williams, G. R. , Tawfik, E. , Zhang, T. , Volitaki, C. , Craig, D. Q. M. , Healy, J. , & Phillips, J. B. (2020). Controlled local release of PPARγ agonists from biomaterials to treat peripheral nerve injury. Journal of Neural Engineering, 17(4), 046030. 10.1088/1741-2552/aba7cc 32780719

[wsbm1593-bib-0048] Rayner, M. L. D. , Healy, J. , & Phillips, J. B. (2020). Drug therapies for peripheral nerve injuries. In Peripheral nerve tissue engineering and regeneration (pp. 1–27). Springer International Publishing. 10.1007/978-3-030-06217-0_16-1

[wsbm1593-bib-0049] Rayner, M. L. D. , Laranjeira, S. , Evans, R. E. , Shipley, R. J. , Healy, J. , & Phillips, J. B. (2018). Developing an In vitro model to screen drugs for nerve regeneration. Anatomical Record, 301(10), 1628–1637. 10.1002/ar.23918 PMC6282521 30334365

[wsbm1593-bib-0050] Rechthand, E. , Smith, Q. R. , & Rapoport, S. I. (1988). A compartmental analysis of solute transfer and exchange across blood‐nerve barrier. American Journal of Physiology, 255(2), R317–R325. 10.1152/ajpregu.1988.255.2.r317 2457330

[wsbm1593-bib-0051] Rittner, H. L. , Amasheh, S. , Moshourab, R. , Hackel, D. , Yamdeu, R.‐S. , Mousa, S. A. , Fromm, M. , Stein, C. , & Brack, A. (2012). Modulation of tight junction proteins in the perineurium to facilitate peripheral opioid analgesia. Anesthesiology, 116(6), 1323–1334. 10.1097/ALN.0b013e318256eeeb 22534246

[wsbm1593-bib-0052] Rosner, B. I. , Siegel, R. A. , Grosberg, A. , & Tranquillo, R. T. (2003). Rational design of contact guiding, neurotrophic matrices for peripheral nerve regeneration. Annals of Biomedical Engineering, 31(11), 1383–1401. 10.1114/1.1626118 14758929

[wsbm1593-bib-0053] Rutkowski, G. E. , & Heath, C. A. (2002). Development of a bioartificial nerve graft. I. Design based on a reaction‐diffusion model. Biotechnology Progress, 18(2), 362–372. 10.1021/bp020300f 11934308

[wsbm1593-bib-0054] Sano, Y. , Shimizu, F. , Nakayama, H. , Abe, M. , Maeda, T. , Ohtsuki, S. , Terasaki, T. , Obinata, M. , Ueda, M. , Takahashi, R. , & Kanda, T. (2007). Endothelial cells constituting blood‐nerve barrier have highly specialized characteristics as barrier‐forming cells. Cell Structure and Function, 32(2), 139–147. 10.1247/csf.07015 18057801

[wsbm1593-bib-0055] Shipley, R. J. , Smith, A. F. , Sweeney, P. W. , Pries, A. R. , & Secomb, T. W. (2019). A hybrid discrete–continuum approach for modelling microcirculatory blood flow. Mathematical Medicine and Biology: A Journal of the IMA., 37, 40–57. 10.1093/imammb/dqz006 30892609

[wsbm1593-bib-0056] Stamatelos, S. K. , Kim, E. , Pathak, A. P. , & Popel, A. S. (2014). A bioimage informatics based reconstruction of breast tumor microvasculature with computational blood flow predictions. Microvascular Research, 91, 8–21. 10.1016/j.mvr.2013.12.003 24342178 PMC3977934

[wsbm1593-bib-0057] Standring, S. (2020). The history of nerve repair. In J. Phillips , D. Hercher , & T. Hausner (Eds.), Peripheral nerve tissue engineering and regeneration (pp. 1–32). Springer International Publishing. 10.1007/978-3-030-06217-0_1-2

[wsbm1593-bib-0058] Swabb, E. A. , Wei, J. , & Gullino, P. M. (1974). Diffusion and convection in normal and neoplastic tissues. Cancer Research, 34(10), 2814–2822. 4369924

[wsbm1593-bib-0059] Sweeney, P. W. , d'Esposito, A. , Walker‐Samuel, S. , & Shipley, R. J. (2019). Modelling the transport of fluid through heterogeneous, whole tumours in silico. PLoS Computational Biology, 15(6), e1006751. 10.1371/journal.pcbi.1006751 31226169 PMC6588205

[wsbm1593-bib-0060] Tajdaran, K. , Chan, K. , Shoichet, M. S. , Gordon, T. , & Borschel, G. H. (2019). Local delivery of FK506 to injured peripheral nerve enhances axon regeneration after surgical nerve repair in rats. Acta Biomaterialia, 96, 211–221. 10.1016/j.actbio.2019.05.058 31141732

[wsbm1593-bib-0061] Tajdaran, K. , Shoichet, M. S. , Gordon, T. , & Borschel, G. H. (2015). A novel polymeric drug delivery system for localized and sustained release of tacrolimus (FK506). Biotechnology and Bioengineering, 112(9), 1948–1953. 10.1002/bit.25598 25850693

[wsbm1593-bib-0062] Taylor, C. S. , & Haycock, J. W. (2020). Biomaterials and scaffolds for repair of the peripheral nervous system. In J. Phillips , D. Hercher , & T. Hausner (Eds.), Peripheral nerve tissue engineering and regeneration (pp. 1–35). Springer International Publishing. 10.1007/978-3-030-06217-0_3-1

[wsbm1593-bib-0063] Waters, S. L. , Schumacher, L. J. , & El Haj, A. J. (2021). Regenerative medicine meets mathematical modelling: Developing symbiotic relationships. Npj Regenerative Medicine, 6(1), 24. 10.1038/s41536-021-00134-2 33846347 PMC8042047

[wsbm1593-bib-0064] Wilcox, M. B. , Laranjeira, S. G. , Eriksson, T. M. , Jessen, K. R. , Mirsky, R. , Quick, T. J. , & Phillips, J. B. (2020). Characterising cellular and molecular features of human peripheral nerve degeneration. Acta Neuropathologica Communications, 8(1), 51. 10.1186/s40478-020-00921-w 32303273 PMC7164159

[wsbm1593-bib-0065] Wu, L. , & Brazel, C. S. (2008). Modifying the release of proxyphylline from PVA hydrogels using surface crosslinking. International Journal of Pharmaceutics, 349(1–2), 144–151. 10.1016/j.ijpharm.2007.08.007 17875374

[wsbm1593-bib-0066] Zalewski, A. A. , Kadota, Y. , Azzam, N. A. , & Azzam, R. N. (1993). Observations on the blood and perineurial permeability barriers of surviving nerve allografts in immunodeficient and immunosuppressed rats. Journal of Neurosurgery, 78(5), 794–806. 10.3171/jns.1993.78.5.0794 8468610

[wsbm1593-bib-0067] Zuo, K. J. , Saffari, T. M. , Chan, K. , Shin, A. Y. , & Borschel, G. H. (2020). Systemic and local FK506 (tacrolimus) and its application in peripheral nerve surgery. The Journal of Hand Surgery, 45(8), 759–765. 10.1016/j.jhsa.2020.03.018 32359866

